# Design of Recyclable Plastics with Machine Learning and Genetic Algorithm

**DOI:** 10.1021/acs.jcim.4c01530

**Published:** 2024-12-03

**Authors:** Chureh Atasi, Joseph Kern, Rampi Ramprasad

**Affiliations:** School of Materials Science and Engineering, College of Engineering, Georgia Institute of Technology, 771 Ferst Dr. N.W., Atlanta, Georgia 30318, United States

## Abstract

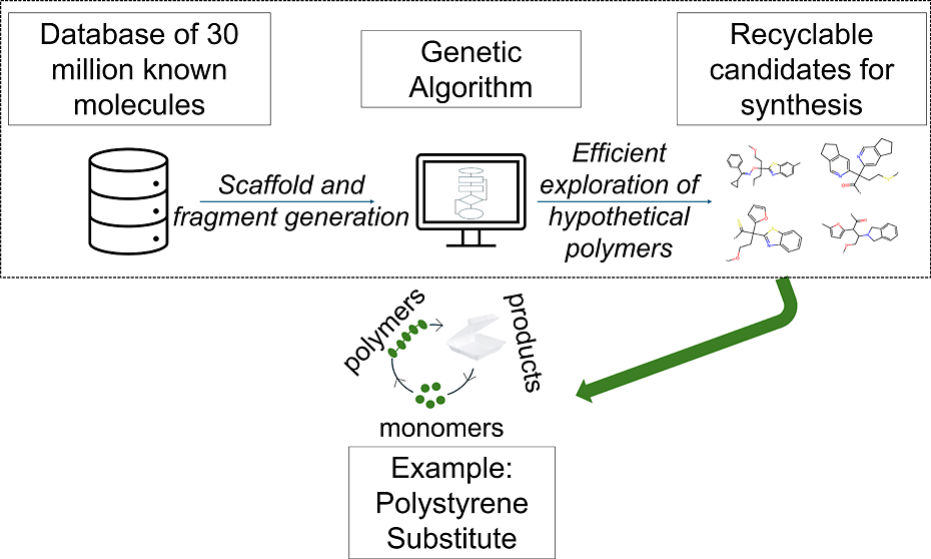

We present an artificial intelligence-guided approach to design durable and chemically recyclable ring-opening polymerization (ROP) class polymers. This approach employs a genetic algorithm (GA) that designs new monomers and then utilizes virtual forward synthesis (VFS) to generate almost a million ROP polymers. Machine learning models to predict thermal, thermodynamic, and mechanical properties—crucial for application-specific performance and recyclability—are used to guide the GA toward optimal polymers. We present potential substitute polymers for polystyrene (PS) that achieve all property targets with low estimated synthetic complexity.

## Introduction

Plastics are integral to modern life, offering versatility and convenience across numerous applications, including packaging, energy reduction, and electronic devices.^1−4^ While plastics offer strength and durability during their useful lifespan, these qualities become significant drawbacks at their end-of-life, with most materials being disposed of in landfills or released into the environment.^5,6^ Unfortunately, this is a problem due to the ever-increasing amounts of plastics being produced.^7^ These synthetic materials persist in the environment for hundreds of years, taking an extraordinarily long time to decompose.^8^ Microplastics, resulting from the breakdown of larger plastic items,^9^ exacerbate the issue by infiltrating the food chain^10^ and accumulating in human bodies.^11^ They also accumulate in landfills,^12^ pollute oceans,^13−15^ and endanger wildlife.^16^

One of the most widely used plastics, polystyrene (PS), contributes significantly to environmental damage through its microplastics.^17,18^ Our focus here lies in developing an alternative material to PS that could be chemically recycled. This choice also stems from the significant presence of PS in both U.S. and European plastic production, as economies of scale are necessary to render recycling economically viable.^8,19,20^ Furthermore, the lack of widespread PS recycling is predominantly due to cost barriers,^21,22^ compounded by the toxicity concerns associated with styrene, the monomer used in PS production, and PS microplastics.^23−25^ To enhance the recyclability of PS and plastics in general, a shift toward more sustainable polymers supporting a circular plastic economy is crucial.^26,27^

Thankfully, the polymer chemical space is expansive, with innumerable viable and environmentally friendly options awaiting discovery. Among them are those with the potential to exhibit desirable properties conducive to recycling without compromising peak performance during usage. However, navigating this vast space presents challenges; traditional Edisonian trial-and-error physical experimentation is slow and expensive. Data-driven techniques and machine learning (ML) have emerged as a powerful alternative paradigm for navigating molecular and polymeric design spaces.^28−34^ Specifically, generative models like variational autoencoders (VAEs) and generative adversarial networks (GANs) have been developed to address design challenges.^35−41^ These models support “inverse design” by mapping a latent space to material properties, enabling the creation of materials with targeted characteristics. Additionally, recent work by Gurnani et al.^42^ introduced a translation-based approach, polyG2G, to generate polymers resistant to dielectric breakdown. Selected polymers from these publications are showcased in Table 1. While effective for producing polymers with specific attributes, these approaches do not connect polymers to their monomer structures—an essential factor for synthesis. This gap underscores the need to combine inverse design algorithms with systems that can develop polymers optimized for chemical recyclability and straightforward synthesis.

**Table 1:**
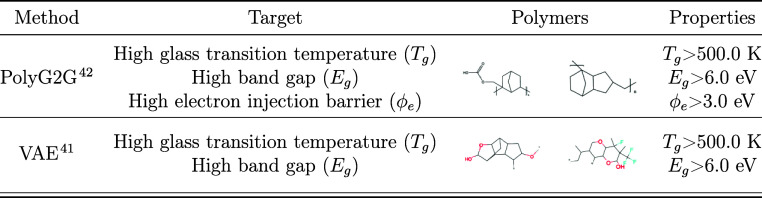
Table Highlighting Previous Successful Examples of Computational Generative Design in Developing Polymers That Meet Specific Target Criteria^a^

a It includes the generative methods employed, the target criteria for the designs, the polymers that met these criteria, and the properties of those polymers.

The genetic algorithm (GA), a type of generative model, stands out as a promising optimization algorithm for conducting efficient searches. It boasts a rich history of application in polymer and molecule design spanning several decades.^43−46^ Notably, the GA excels in navigating vast polymer spaces to swiftly identify optimal designs tailored to specific tasks.^29,47^

This study presents a novel GA tailored for the design of chemically recyclable polymers. Departing from previous methodologies where the focus was on polymer design followed by retrosynthesis techniques to predict monomers,^47,48^ our approach begins with monomer design. In this work, every monomer designed belongs to a specific class that has its associated polymerization pathway (Table 3). The newly designed molecules undergo virtual polymerization through these predefined reaction templates to generate hypothetical polymers. This process is referred to as virtual forward synthesis (VFS). For this study, our focus was exclusively on ring-opening polymerization (ROP) reactions and monomers capable of accommodating them. This choice stems from the significant potential of ROP polymers for chemical recycling into monomers.^49−51^ Additionally, our recyclability modeling requires knowledge of the monomer structure and is only viable for ROP reactions.^52^

In past successful approaches to genetic algorithms, the design criteria only targeted the polymers, without considering the monomer.^29,47^ However, for every polymer proposed, it was difficult to know how to synthesize as there was no starting monomer nor a clear pathway for polymerization (addition, condensation, ROP, etc...) This lack of knowledge of the monomer and reaction class prohibits enthalpy calculation which is indispensable for recyclability measures. By introducing monomers and utilizing a state-of-the-art enthalpy prediction model (see section Methods: design) this work aims to achieve that goal of computationally searching for recyclable polymers.

On the experimental side, there have already been efforts to design recyclable ROP class polymers.^53^ In this paper, they gather and present about 60 polymers as a proof-of-concept in terms of recyclability. Only one polymer could achieve all our design criteria. In fact, considering all literature, only one other polymer meets our design criteria for PS.^54,55^ Both polymers are presented in Figure 2b. While this experimental synthesis demonstrates the potential of ROP class polymers, it does not guarantee that these specific polymers can be produced at scale. As such it is paramount to explore the magnitude of other candidates to increase the chances of finding a polymer that could eventually solve our global problem at scale. Our model can produce thousands of potential recyclable polymers that achieve design targets.

In the following sections, we outline the process of finding new chemically recyclable polymers that meet performance criteria, like thermal stability, high stiffness and strength, and effective thermal insulation.

## Methods

To guide the GA toward the desirable molecules and their polymers, ML models are employed to predict key properties of polymers such as *T*_g_, decomposition temperature (*T*_d_), tensile strength at break (σ_b_), Young’s modulus (*E*), and heat capacity (*C*_p_). These properties, along with their desired target values that lead to PS substitutes are listed in Table 2. The synthetic complexity of each novel molecule is optimized too, increasing the likelihood of creating synthesizable designs. Therefore, on the whole, the GA streamlines the process of identifying suitable polymers by leveraging rapid property predictions using ML models.

**Table 2:** Design Objectives for PS Replacement, Surrogate Properties Correlated with These Objectives, and Their Target Values Used During the Genetic Algorithm Design Process

property	target	goal
*T*_g_	>373 K	thermal stability
*T*_d_	>473 K	thermal stability
σ_b_	>39 MPa	strength
*E*	>2 GPa	rigidity
*C*_p_	>1.24 J/gK	thermal insulation
Δ*H*	>−10, <−20 kJ/mol	chemical recyclability
SA score	<3	monomer synthetic complexity

Recyclability is evaluated through the enthalpy calculations. Enthalpy is chosen because we have modeling capabilities to predict the enthalpy of polymerization, and it is proportional to the ceiling temperature (*T*_c_). The *T*_c_, defined as the enthalpy of polymerization over the entropy of polymerization, determines the temperature above which monomers are more stable, triggering polymer depolymerization. By designing polymers with a *T*_c_ below the *T*_d_, we can ensure the polymers are chemically recyclable.

Although predictive methods for *T*_c_ and entropy are lacking, enthalpy predictions are feasible.^52^ These predictions necessitate ROP polymers, focusing our design space on this area. This ROP design space has also previously been identified in literature as promising for chemical recyclability^49−51^). Specific enthalpy design criteria are defined in section Methods: design.

After identifying desirable polymers based on all of their properties, their chemistry is further evaluated to ensure they can be synthesized. Additionally, the ROP classes to which these desirable polymers belong are examined. The goal is to create polymers with specific usage properties that are chemically recyclable, allowing them to be broken down back into their original monomers for reuse, thereby promoting sustainability.

### Genetic Algorithm Implementation

**Figure 1:**
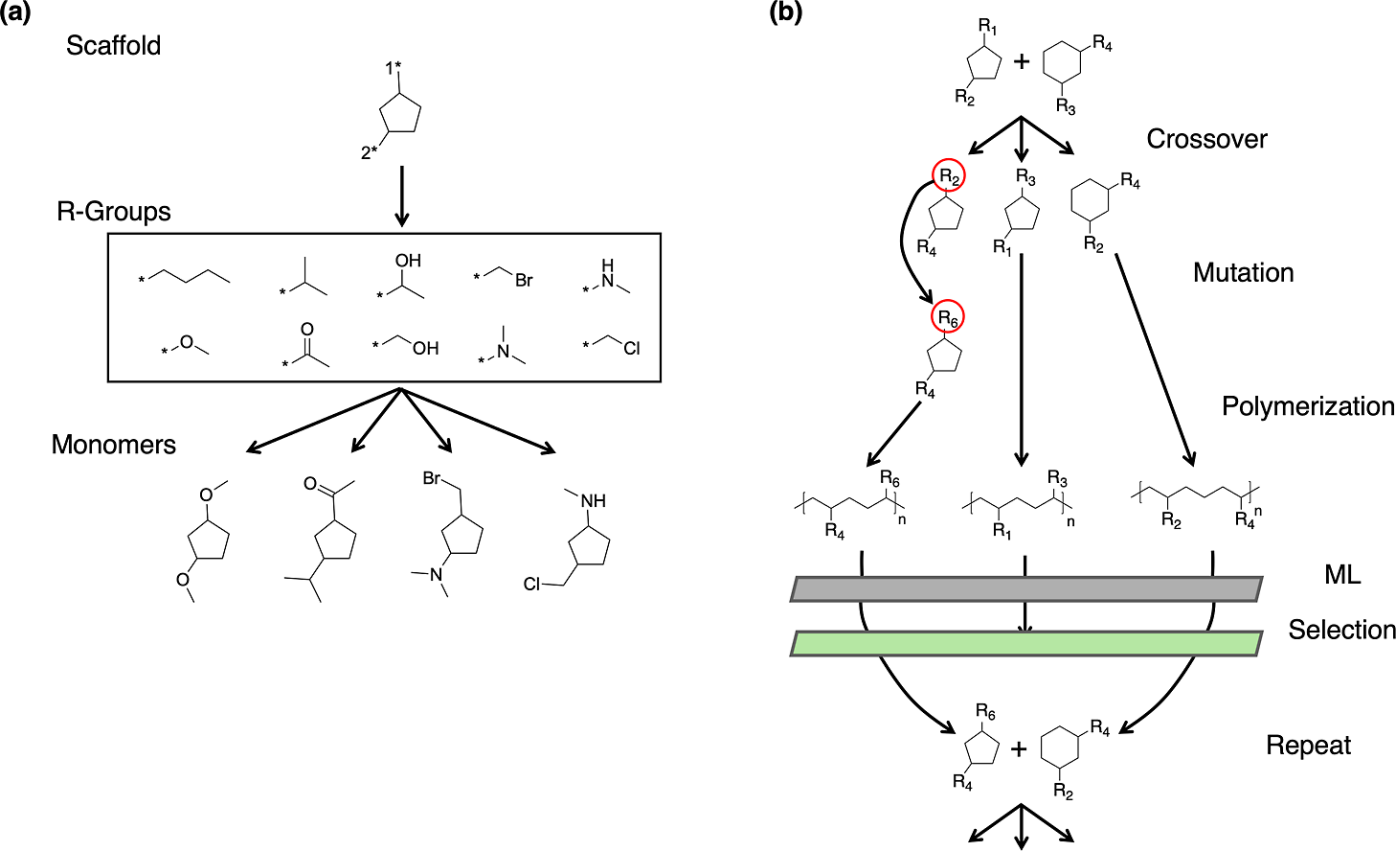
(a) Example of GA initialization. The scaffold is a five-member cycloalkane with functionalization positions denoted by the labeled asterisks (*). New molecules are generated by functionalizing these locations with the corresponding R-groups displayed in the middle. The R-groups are then randomly attached to the scaffolds to form several monomers. Four examples of such monomers are shown at the bottom, each consisting of the base scaffold and two R-groups. Subsequently, the newly formed monomers undergo VFS. (b) Schematic illustration of the GA workflow, depicting the key processes of crossover, mutation, polymerization, property prediction, and fitness evaluation/selection.

The GA, with the aid of the python package RDKIT,^56^ designs hypothetical monomers, which are subsequently subjected to virtual reaction templates generating novel polymers. The initialization of this process involves the following steps, as exemplified in Figure 1a:

1. Scaffold generation: a molecular scaffold is constructed (Figure 1a “Scaffold”), comprising a base structure capable of polymerization and featuring variable functionalization sites.
2. R-group creation: a global list of R-groups (Figure 1a “R-Groups”) is created, serving as chromosomes for the GA’s optimization process.
3. Molecule creation: these R-groups are bonded to the scaffolds using the atoms adjacent to the asterisks (see * in Figure 1a “Scaffold” & “R-Groups”) to create molecules that can be then virtually polymerized.
4. Virtual polymerization: SMiles ARbitrary Target Specification (SMARTS) patterns, which are line notations used to define reaction templates, are defined to virtually polymerize the molecule using VFS (Figure 1b “Polymerization”). This generates polymers suitable for ML property prediction.

Following this, the GA creates an initial population by generating *n* molecules from the scaffold and randomly assigning R-groups from the global list to their functionalization positions. Each newly formed molecule undergoes polymerization, and the resulting polymer’s properties (section Methods: design) are predicted using the ML models. The polymers are then ranked according to the fitness function outlined in section Methods: fitness, enabling the selection of top-ranked polymers as “parents” for the subsequent generation.

The parent polymers are randomly paired and generate a predetermined number of children. Each child polymer randomly inherits one of its parents’ scaffolds, and then crossover occurs, combining the parents’ R-groups into a pool. The child’s functionalization sites are then randomly assigned R-groups from this pool. Subsequently, a small subset of child polymers undergo mutation, where some functionalization sites are replaced with random R-groups from the global list. If a child polymer has been previously encountered in a prior generation, mutation continues until a unique molecule is generated. This generation of polymers is then fingerprinted^28^ and undergoes property prediction using ML. The topmost desirable polymers of that generation become the “parents”. This iterative process, illustrated in Figure 1b, repeats for a set number of generations, driving the evolution of new polymers.

### Design Target and Property Predictors

PS, commonly employed in take-out containers and packaging, displays exceptional performance, encompassing thermal stability, high stiffness and strength, and good thermal insulation. Key properties influencing these characteristics include *T*_g_, *T*_d_, σ_b_, *E*, and *C*_p_. Properties that dictate chemical recyclability for a replacement polymer include the enthalpy of polymerization (Δ*H*). Accordingly, we adopt these properties as design targets that govern the fitness function of the GA, with each property’s target value and its purpose detailed in Table 2.

*T*_g_ must exceed the boiling point of water (373 K) to ensure the new polymer remains stable when exposed to heat. Setting *T*_d_ 100 K above this value prevents decomposition during chemical recycling.

To ensure durability, σ_b_ surpassing 39 MPa and E exceeding 2 GPa are selected to prevent breakage and excessive bending, aligning with the properties of PS.^57^ Additionally, *C*_p_ equal to that of PS is adopted to prevent burns from hot contents.

In contrast to other properties where surpassing a threshold is the goal, we aim for the Δ*H* to fall within the range of −10 to −20 kJ/mol. If Δ*H* is too negative, the *T*_c_ will likely be too high, and polymers will not be depolymerizable before decomposition. However, if it is too close to zero, The *T*_c_ will likely be too low and the polymer will not be useable at room temperature.

These properties are predicted using two subsets of previously developed and published models: a Gaussian process regression (GPR) model to predict Δ*H* based on the polymer and monomer,^58^ and a multitask neural network (MTNN) trained on homo and copolymer data to predict all other properties.^59^ These ML models have been developed and extensively tested on a data set comprising both previously synthesized polymers and hypothetical polymers with properties predicted using density functional theory (DFT) and molecular dynamics (MD). Specifically, we highlight several previous studies that have combined DFT-calculated values with ML techniques and chemical intuition to achieve more accurate results.^60,61^ Further details pertaining to the models used in this study, including training data used, algorithmic details, and accuracy can be found in their respective publications.^58,59^ A short section regarding these models and their requisite polymer fingerprints is provided in the Supporting Information Section 1 “ML Models and Fingerprinting”.

Finally, a synthetic feasibility target criterion is established to reduce the complexity of designed molecules. The synthetic accessibility score (SA score) method, which uses a combination of fragment contributions and complexity penalty,^62^ is used to calculate molecular complexity. The SA score ranges from 1 to 10, where 1 indicates a molecule is more likely to be synthesized and 10 indicates it is less likely to. The target value is set to 3, a common value for synthetic molecules.^62^ To design novel polymers, aiming for monomers with a low SA score (<3) will increase their chances of being synthesized.

### Fitness Assessment

Underpinning any GA is the fitness function it optimizes over. We use a clamping fitness function that has previously performed well for many-property GA optimizations.^47^ However, as the target values for Δ*H* fall within a range, the predicted values must first be transformed to facilitate scoring. This fitness assessment is defined as follows:

1. Enthalpy transformation: Δ*H* values are transformed so that the transformed Δ*H* target is in the same range as the other property targets, enabling the application of standard clamping across properties and Min–Max scaling. The revised objective is to attain a Δ*H* greater than 10 kJ/mol. For any polymer *i* the enthalpy transformation is defined in eq 1

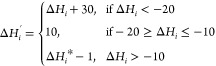
1

2. SA score transformation: since we aim to minimize this target criterion and are using a MinMaxScaler that prioritizes higher values, we take the additive inverse of the target criterion to optimize this property

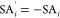
2

**Figure 2:**
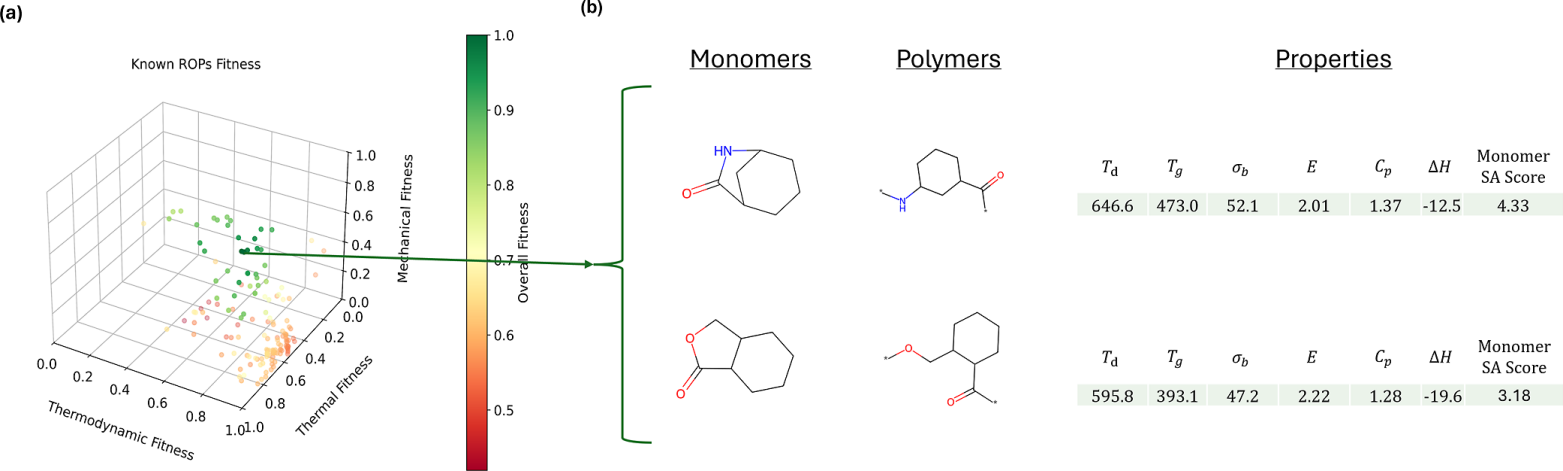
(a) Three-dimensional scatter plot displaying the predicted thermal (*T*_g_, *T*_d_), mechanical (σ_b_, *E*), and thermodynamic (*C*_p_, Δ*H*) property values of known ROP polymers. Predicted values are scaled according to section Methods: fitness and averaged by property category to facilitate visualization of the six properties in three dimensions. The colors represent the overall fitness, found by averaging the scaled values for all properties. (b) Some of the already known and synthesized polymers^54,55^ are predicted to have achieved the first 6 target criteria.

3. Clamping of predicted properties: predicted properties exceeding the target thresholds were clamped to the targets using eq 3

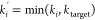
3*k*_*i*_ represents the predicted and transformed values of property *k* for polymer *i*. *k*_target_ denotes target value as defined in Figure 2a. *k*_*i*_^′^ signifies the clamped predicted value of polymer *i*. This transformation prioritizes polymers that satisfy all criteria over those that excel in only a few.

4. Normalization and Fitness Calculation: The clamped property values are normalized within the range of 0 to 1 using a MinMaxScaler. A fitness value for each polymer is then calculated by adding these normalized properties, as described in eq 4

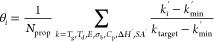
4here, *k*_min_^′^ represents the minimum clamped predicted value for all polymers in the data set. *N*_prop_ is the total number of properties being optimized for. θ_*i*_ represents the fitness score for polymer *i*. Each property receives an equal weight in the fitness calculation.

We note that, in section Results and Discussion, SA Score is not used to screen for promising candidates because molecules that obtain an SA Score higher than 3 can still be synthesized. Hence “desired” polymers need only achieve the first 6 properties—*T*_d_, *T*_g_, σ_b_, E, Δ*H*, *C*_p_.

Figure 2a displays a three-dimensional scatter plot of the predicted property fitness values for known ROP polymers, categorized into thermal (*T*_g_, *T*_d_), mechanical (σ_b_, *E*), and thermodynamic (*C*_p_, Δ*H*) properties. The colors represent the overall fitness, calculated using eq 4, while each position along the axis corresponds to a modified version of eq 4 where fitness is averaged by category rather than all properties. The plot reveals that only two out of all known ROP polymers meet the six target property criteria, underscoring the need for improved methods to explore the polymer space for recyclable candidates. Figure 2b highlights these two previously synthesized polymers. The first monomer is a hybrid that combines a 5-membered lactam, known for its low ceiling temperature, to enhance chemical recyclability, with a 7-membered ring lactam, which has a high ceiling temperature, to achieve strong thermomechanical properties.^54^ The second monomer incorporates a 5-membered γ-butyrolactone for its recyclability benefits but includes a trans-fused cyclohexyl ring to provide the necessary physical properties without hindering depolymerization processes.^55^ The relatively high monomer SA Score indicates that monomers with values above 3 can indeed be synthesized. All in all, the scarcity of known polymers meeting these targets motivates further search.

### Compound Validation: Scaffolds, R-Groups, and Reactions

#### Scaffolds

We leverage a comprehensive data set of 30 million known compounds to enhance the likelihood that the GA-generated molecules are valid. A key part of our method is selecting the top 4–5 scaffolds associated with the most frequently observed molecules in existing literature. These scaffolds were generated by searching a vast database of approximately 30 million known molecules for structures matching those in the “Reaction” column of Table 3 and identifying the most common combinations of R-group locations in the rings. The data set was compiled from five diverse sources: ZINC15, ChemBL, literature-derived compounds, an eMolecules database dump from December 19, 2020, and data scraped from a VWR database.^63−66^ These scaffolds represent the most encountered structures which increases the likelihood that molecules designed from them align closely with existing chemical structures. As such we believe that the molecules these scaffolds form are already likely to be synthesizable. Furthermore, to simplify the molecules resulting from the GA, we deliberately selected scaffolds with only one or two available R-group attachment points. Additional details on the scaffold selection process can be found in the Supporting Information Section 2 “Scaffold Generation”. As shown in Table 3, we investigated a total of 8 ROP classes encompassing 37 distinct scaffolds.

**Table 3:**
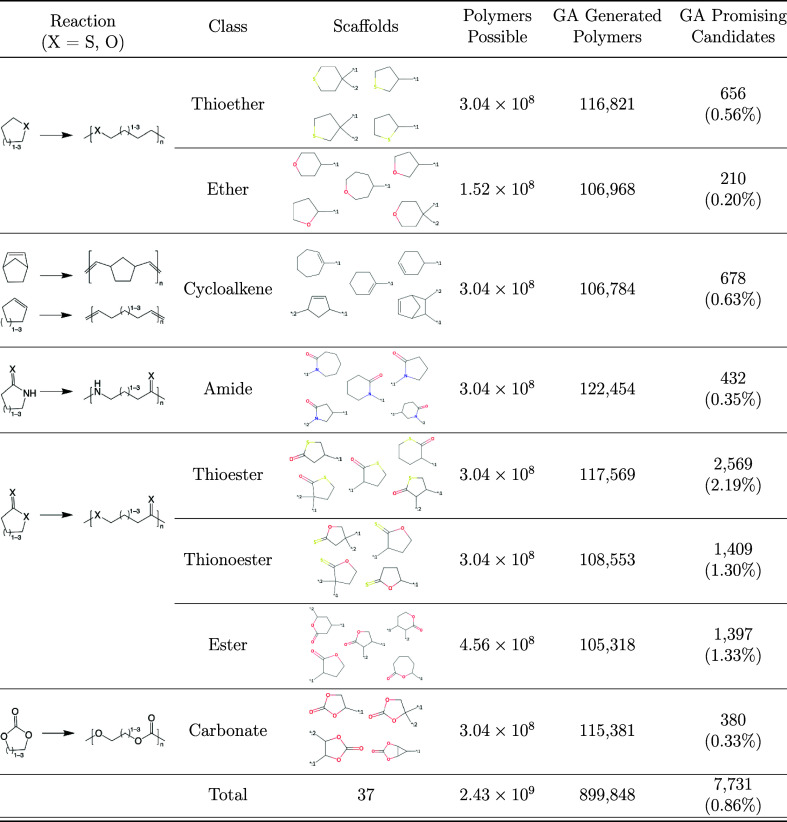
Table Displaying the Simplified ROP Virtual Reaction the Molecule Undergoes, the Class of Ring That was Opened, the Scaffolds Used During a GA Run, the Total Number of Possible Polymers from All Combinations of Adding Our R-Groups to the Scaffolds, the Number of Polymers Generated During the GA Runs, and the Number That Met All Screening Criteria (Except SA Score) for the Specific Class^a^

a For the promising candidates, all polymers with single (N–N) nitrogen bonds were excluded.

#### R-Groups

To create the potential R-groups for attachment to the scaffolds, we conducted an exhaustive search of the database for all molecules amenable to being opened via one of the specified reaction classes. Approximately 3.4 million molecules met this criteria. Subsequently, we employed breaking of retrosynthetically interesting chemical substructures (BRICS) decomposition on these molecules, enabling us to isolate fragments possessing a single connection point compatible with the chemical context of the scaffolds. This process allowed us to identify 29,030 fragments that could interface with the chemical environment of the scaffolds, facilitating their integration into the desired molecular structures. This process enhances the chemical validity of our compounds, aligning them with established molecular guidelines from the literature. While using BRICS might constrain the chemical space, as all generated molecules would only exhibit connections that are known to exist in previously synthesized molecules, it does help in increasing chemical validity. Further refining our selection, we excluded groups featuring alcohols, primary and secondary amines, carboxylic acids, carbonic acids, carbamic acids, and acidic methylene groups–highly reactive functional groups that could complicate polymerization. Additionally, we removed duplicate R-groups with variations in stereochemistry, as these aspects are not adequately handled by our ML models. This process culminated in a curated list of 12,329 R-groups.^67^

#### Reactions

Once the molecule is created, then we use the known reaction pathways^68^ outlined in Table 3 to turn these monomers into potentially valid polymers to be fed into the ML model. Table 3 presents the list of reactions, the class of monomers opened in the reaction, along with the scaffolds used for a molecule during a run of the GA, the total number of different combinations that can be created by attaching all the R-groups to the scaffolds, the total number of polymers generated by the GA, and the number of polymers found by the GA that meet all property criterion.

#### GA Runs

For every monomer class, we conducted three iterations of the GA, as multiple iterations have been demonstrated as one of the most effective means to enhance molecule diversity.^47^ Initially, a population of 300 molecules was randomly generated. In each subsequent generation, the first 100 top-performing polymers, identified through their fitness function, were chosen. These top polymers were then randomly paired to form 200 families, capable of producing up to three offspring each, although fewer were generated if no unique combinations were feasible. Approximately 7.5% of these child polymers would have one R-group mutated. Moreover, to enrich the diversity of generated polymers, each polymer’s simplified molecular-input line-entry system (SMILES) representation was stored in a Python set. If a previously encountered SMILES was identified, the corresponding monomer underwent mutation until a unique polymer emerged.

## Results and Discussion

**Figure 3:**
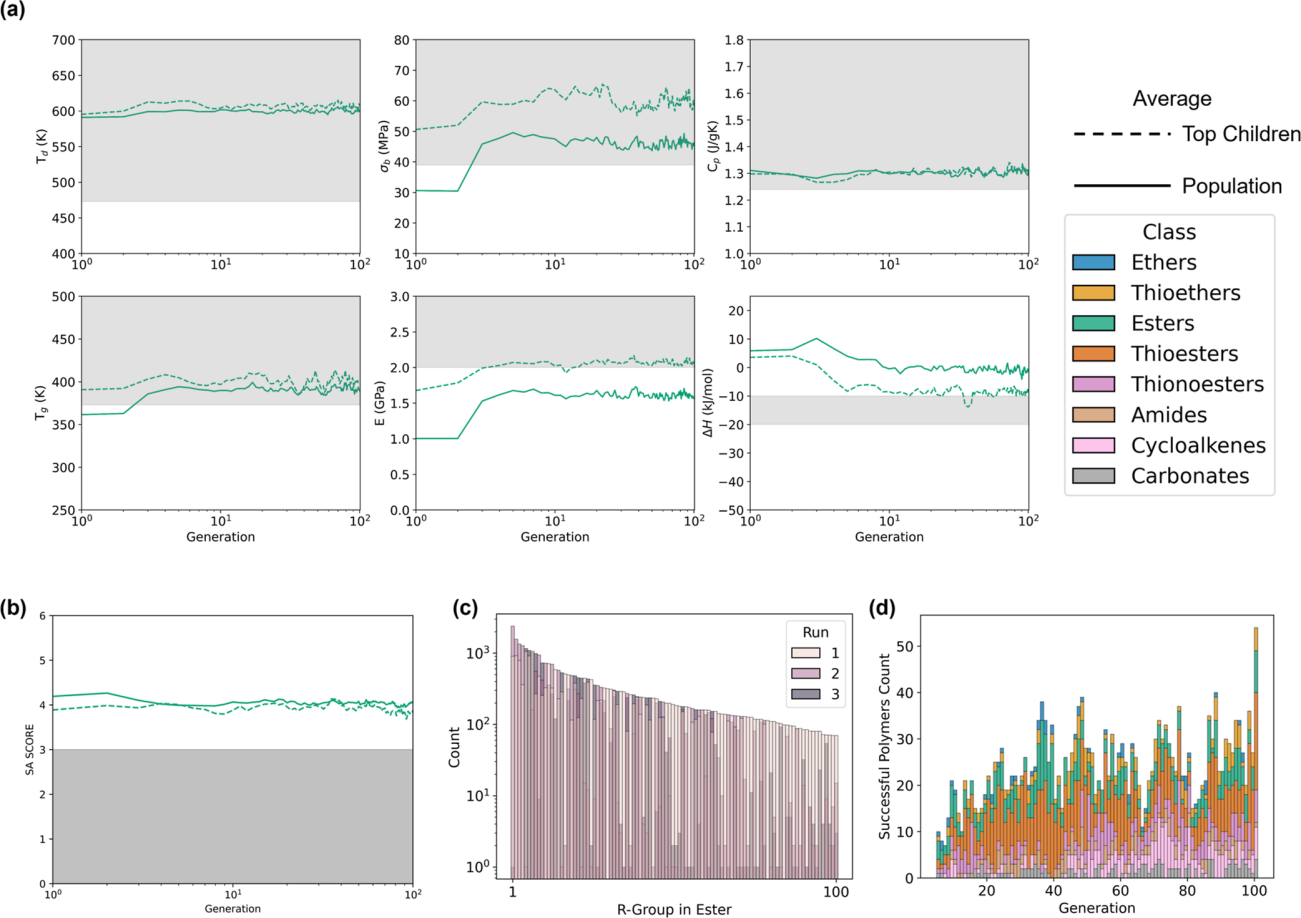
(a) Line plot showing the change in average properties for top children polymers (dashed lines) and the total population (solid lines) over generations for one run of the GA on the ester class of monomers. The top 100 polymers, selected based on their fitness function values, serve as parents for the next generation. The gray region indicates the target property range. (b) Line plot demonstrating the static evolution of the SA Score through generations for the ester class of monomers. (c) Layered bar plot comparing the frequency of the most common R-groups in run 1 of the ester scaffolds with runs 2 and 3. (d) Stacked histograms display the count of polymers meeting all target properties per generation for the run in (a).

Figure 3a illustrates the progression of polymer properties across generations within one GA run for esters, with shaded areas denoting the desired ranges for these properties. The choice of esters stems from its ability to best depict trends in the evolution of the GA polymers over time. Initial population averages for *T*_d_ and *C*_p_ fortuitously fall within these ranges, while the top 100 children polymers, achieve the desired *T*_g_ and σ_b_. Consequently, subsequent generations swiftly converge toward these targets. Across the next ten generations, the GA refines *E* and Δ*H*, culminating in top children polymer averages getting close to achieving these objectives while still adhering to the design objectives for the other properties.

The gap between parental and population averages arises due to the deliberate selection of top-performing polymers as parents. The next generation generated by crossover and mutation of these parents is not guaranteed to have superior properties as children, hence why the population averages remain distant. Conversely, when this gap diminishes, it typically indicates that the property in question is no longer the focus of optimization, as its desired threshold has already been attained as seen for *T*_d_. Thus, parent polymers are no longer selected based on their superior performance in these properties and their average values remain close to the population average. This is not the case for σ_b_ though, likely because it is correlated with *E*, and *E* is still being optimized for.

Despite the overall progress, the top children averages barely reach *E* and Δ*H* targets, and population averages remain even further away. Moreover, top-performing children from other ROP classes never quite reach the threshold (see Supporting Information Figure S7). This result can be partly attributed to the enforced mutation mechanism when encountering previously identified molecules. Earlier studies demonstrated that a high mutation rate can hinder a GA’s ability to optimize its objective.^47^ This is particularly problematic in runs with a large number of scaffolds with a single R-group location, where child polymers created through crossover are likely to have been previously encountered especially if both parents also have a single R-group location. In such cases, the child polymer is forced to mutate with a random R-group from the global list to avoid duplicating the parent structure, and its new structure will not be optimized for the target properties. In contrast, scaffolds with multiple R-group locations, such as esters, are more resilient to forced mutation, offering a wider range of potential polymers, thus reducing the likelihood of previous encounters. Even if forcibly mutated, they are likely to retain some optimized R-groups, ensuring a better chance of retaining target properties. This could explain why ethers, cycloalkenes, and amides tend to have the fewest promising candidates (see Table 3) since they have the largest number of scaffolds with only one R-group location (see Supporting Information Figure S3).

Another possible explanation is that the GA simply did not run long enough. As observed in previous studies,^47^ properties often remain stagnant until a mutation occurs that suddenly and dramatically improves the desired property, a phenomenon known as punctuated equilibrium.^69^ This effect is evident near generation 40 of esters, where a sudden drop in Δ*H* occurs. However, continuing runs for many generations can be problematic, as GAs tend to get “stuck” once they find optimized zones (local minima), resulting in low diversity of new solutions. To mitigate this, restarting with a new, randomly generated initial population can be beneficial.^47^ As shown in the layered bar chart in Figure 3c, the most common R-group in one GA run, likely kept due to its effectiveness at solving the properties, is often significantly different in the next run, and by restarting, new and unique solutions, can be found.

To illustrate the molecular complexity, the evolution of the SA Score is depicted in Figure 3b. Notably, the average values for the parents and population remain stagnant during the run for the ester class (see Supporting Information Figure S7 for the rest of the ROP classes). This suggests a trade-off exists between reducing the molecular complexity of the monomers and finding polymers that meet all mechanical, thermal, and thermodynamic requirements. As such, across all runs, only one “ideal” polymer was found that satisfies all 6 properties as well as our reduced complexity. However, the GA has succeeded in generating 60 desired polymers with slightly higher SA Score values (≤3.5).

Ignoring SA Score, as even those monomers with high scores may be synthesizable, 7731 polymers meet the other six properties: *T*_d_, *T*_g_, σ_b_, *E*, Δ*H*, and *C*_p_. We refer to these as “desired” polymers. Despite these properties not being met on average during the GA runs in Figure 3a, numerous solutions for polymers attaining all 6 properties, were found at each generation, as shown in Figure 3d. Initially, few polymers met all 6 desired properties, but the runs quickly optimized to find unique solutions consistently across generations. The GA’s ability to continue to find novel solutions is attributed to the mutations discussed earlier, which ensures that new molecular spaces continue to be explored. Notably, most polymers that achieved all properties had two R-group locations, as seen in Supporting Information Figure S6a. This also helps explain why runs with fewer scaffolds containing 2 R-group sites have lower counts in Figure 3d. However, increasing the number of R-group locations results in increased molecular complexity, as seen in Figure S6b, again highlighting a trade-off between property optimization and molecular complexity.

**Figure 4:**
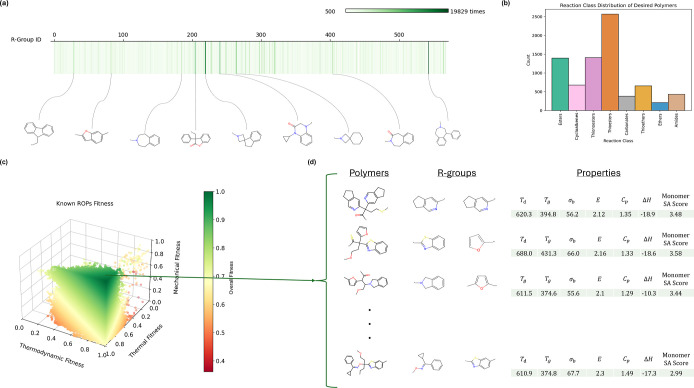
(a) Gene strip demonstrates the overall occurrence of all R-groups with counts of >500 over 100 generations of evolution. Nine top R-groups are indicated using their SMILES representation along with their frequency. (b) The distribution of the reaction classes for the polymers that achieve all properties except SA Score. (c) 3D scatter plot representing Heat, Mechanical, and Thermal fitness on a scale from 0 to 1. The green points are more favorable than the redder points. A total of 7731 desirable polymers have been produced by the GA (d) A collection of generated polymers along with their R-groups that achieve target criteria and possess low molecular SA scores.

In Figure 4a, we present a gene strip highlighting some of the most frequently employed R-groups. Notably, many of the most abundant R-groups form bonds at the cyclic nitrogen, yielding tertiary amines in the monomers. Cyclic structures in general were very common, making up the majority of the most common R-groups. This is likely due to the fact that these chains introduce steric hindrance, constraining chain mobility and enhancing thermal and mechanical properties as a consequence.

Additionally, an ROP class distribution plot, depicted in Figure 4b, illustrates the most effective reaction classes that meet our criteria. Among these, esters and their variations—such as thioesters and thionoesters—are notably the most prevalent. Esters stand out with the highest average overall fitness value, and thioesters, in particular, yield the most desirable properties. However, the SA Score for esters are also the highest, indicating they are more challenging to synthesize. This highlights a recurring trade-off between molecular complexity and other technical properties. Given that all seven properties listed in Table 2 are weighted equally, molecular complexity contributes only one-seventh to the overall fitness value. This explains the high overall fitness scores for esters despite the higher complexity. In future studies, we could consider increasing the weight of molecular complexity to identify classes that yield both more feasible and robust materials.

In contrast to the mere two known polymers predicted to achieve all 6 physical properties, as shown in Figure 2b, the GA was capable of finding ∼8k polymers achieving them. The 3D scatter plot in Figure 4c illustrates this, with a dense concentration of green dots representing the numerous polymers meeting all property criteria. Notably, an exhaustive enumerative approach to polymer design would have entailed computing almost 2.5 billion polymers, a nontrivial task. Our GA, however, reduces calculations by a remarkable 99.96%. Furthermore, within the Supporting Information Section titled “Ether Case Study”, we conducted a comparison between the GA and an enumerative approach using the ether scaffolds and 700 R-groups. Through this investigation, we discovered that although none of the 492,800 ethers generated via enumeration fulfilled all the required properties, the GA successfully identified 975 out of the 1241 top 0.5% of polymers, after examining only 38,479 polymers - 7.8% of the total number of polymers possible. This represents a remarkable order of magnitude reduction in search, while still capturing 79% of promising designs. Such results underscore not only the validity but also the efficiency of our GA in navigating complex solution spaces.

Despite the technique’s potential, some limitations remain. Even though SA Score was one of the target criteria, very few polymers attained this target. Among the polymers deemed desirable (those meeting all other criteria), only one met the SA Score target, reported in Figure 4d. As shown in Supporting Information Figure S6c, a significant number of the 7500+ promising candidates exhibit a high SA Score (>3), indicating increased molecular complexity.^70^ Most lab-synthesized molecules have an SA Score of less than 3, while naturally occurring molecules range from 3 to 9 and peak at 6^70^ due to their greater complexity. Thus, because GA molecules will be synthesized in the lab, lower complexities are desired. This highlights the need for further refinements to the GA to prioritize synthetic feasibility. This involves prioritizing less complex scaffolds and R-groups to ensure the chemistry space contains a greater number of potentially producible molecules for the GA to discover. In Figure 4d, we select polymer candidates to replace PS that meet all target criteria and lie in the lower end of the SA Score range.

Previous work on ROP VFS for PS substitutes that is focused on polymer validity was conducted by Kern et al.^71^ The research specifically targets already-known monomers from literature and commercial databases and then polymerizes them through a similar VFS scheme into polymers, filtering the polymers that have attained the properties needed for PS. Their method has provided about 37,000 polymers from previously seen monomers with (SA scores < 7). Our approach complements this work by introducing an intelligent exploration strategy for noncommercially available monomers, tackling a vastly larger search space that would be impractical to navigate using the prior works brute force method.

Overall, the GA significantly streamlines calculation times compared to exhaustive enumerative methods. We can effectively explore the polymer space by targeting only the highest-performing polymers, as defined by user-defined properties, instead of iterating over every single combination. In this study, the GA identified over 7500 polymers with desired mechanical, thermal, and thermodynamic properties while only surveying (fingerprinting + predicting) ∼ 900,000 polymers, which represents a mere 0.037% of the total possible combinations. Additionally, in a small-scale test study (Supporting Information Section 3), the GA captured 79% of promising designs compared to an exhaustive enumerative approach. These findings underscore the effectiveness of the GA in rapidly identifying promising polymer designs, establishing it as a valuable tool in polymer research. This work lays the foundation for other scientists to tackle the specific problem of surveying unknown polymers to find recyclable polymers for any purpose through cheap computational techniques.

## Conclusion

In conclusion, our study introduces a novel genetic algorithm specifically designed to create monomers that can be polymerized into chemically recyclable alternatives to PS. The persistence of PS in our ecosystems, due to its limited recyclability and associated toxicity, underscores the urgency of our research in providing sustainable solutions. By integrating cutting-edge computational design methodologies, such as the utilization of molecular scaffolds, BRICS-derived fragments, advanced VFS techniques, and state-of-the-art ML models, we efficiently traverse molecular design landscapes of billions of hypothetical polymers. Combined with our GA, these approaches help pinpoint over 7500 potential substitute polymers that exhibit the requisite thermal, mechanical, and thermodynamic properties necessary for serving as recyclable alternatives to PS. Noteworthy among these are molecular motifs featuring tertiary amine linkages to the polymer’s backbone and cyclic side chains, likely attributed to their capacity for impeding chain mobility, thereby enhancing thermal and mechanical characteristics. These findings underscore the efficacy of our GA in generating a diverse spectrum of polymers that fulfill the prescribed property criteria, all while significantly mitigating computational overhead compared to exhaustive enumerative methodologies.

One significant limitation of this method is that while the monomer-to-polymer reaction is well-defined, the monomer synthesis pathway remains unclear. Although the SA Score provides insight into synthetic complexity, it does not offer a specific synthesis route for the monomer. To address this, future efforts should prioritize resolving this issue by leveraging retrosynthesis planning tools or integrating established reaction pathways for designing and functionalizing monomers into the GA.^72^ Strategically utilizing established pathways rather than relying solely on BRICS fragments would likely improve monomer synthesis planning and reduce the synthetic complexity of candidate monomers. Importantly, the current GA iteration can support this approach with minor modifications.

## Data Availability

The package, which includes the runner files, open-source code, and postprocessing scripts, is available on GitHub. Additionally, data on the scaffolds and promising polymers identified during our run are also presented on GitHub. We share the csv with the scaffolds found using our algorithm, the counts of unique molecules and the code used to find these scaffolds. The open-source code or package is accessible at https://github.com/Ramprasad-Group/pvfsga. The data is available on GitHub in our group polyVERSE repo.

## Supporting Information Available

We have included a supplementary document that supports the GA with test cases and provides a detailed discussion on the acquisition of molecular scaffolds and R-groups. It also elaborates on the mechanisms of the ML models and fingerprint techniques. The test run demonstrates the GA’s efficiency compared to an exhaustive brute force method for the same input. Additionally, we offer further information about the promising candidates, focusing on their molecular complexity and the number of R-groups. Supplementary: design of recyclable plastics with machine learning and genetic algorithm (PDF)
