# Supplementary material for: Design of Recyclable Plastics with Machine Learning and Genetic Algorithm
Source: J Chem Inf Model. 2024 Dec 3;64(24):9249–59. doi: 10.1021/acs.jcim.4c01530 (PMC11683875; doi:10.1021/acs.jcim.4c01530)
Supplement: Supplementary file 1 — ci4c01530_si_001.pdf [file ci4c01530_si_001.pdf]

Supplementary: Design of Recyclable Plastics with Machine Learning and Genetic Algorithm

Chureh Atasi,* Joseph Kern,* and Rampi Ramprasad*

School of Materials Science and Engineering, College of Engineering,Georgia Institute of Technology, 771 Ferst Dr. N.W., Atlanta, 30318, GA, U.S.A

E-mail: catasi3@gatech.edu; jkern34@gatech.edu; rampi.ramprasad@mse.gatech.edu

ML Models and Fingerprinting

In order to predict the properties of polymers as displayed in Figure 1 (b), the polymers are run through two different Machine Learning Models. For predicting enthalpy of polymerization (ΔH) of the polymers, a Gaussian process regression (GPR) is used. While the rest of the properties ($T_g, T_d, C_p, \sigma_b, E$) are predicted using the multitask neural network (MTNN) models.

Enthalpy of Ring-Opening Polymerization: Gaussian Process Regression

The GPR model's training incorporated a mix of experimental ΔH values from ring-opening polymerization (ROP) polymers and density functional theory (DFT) data. This blended dataset excels in predicting experimental ΔH values for ROP polymers.[1,2] GPR was chosen due to its established reliability in accurately forecasting polymer properties, even with limited data.

Multitask Neural Network

A range of MTNN models utilized diverse data from both homopolymers and copolymers. The training encompassed various correlated property classes, including thermal, mechanical, gas permeability, thermodynamic and physical, electronic, and optical and dielectric properties. The primary focus, illustrated in Table 2, was on thermal, mechanical, and thermodynamic properties. For an in-depth explanation of the training and testing methods, please refer to the original MTNN paper.[3]

Fingerprinting

Polymer fingerprinting is a technique that employs three hierarchical levels of descriptors to represent the structural features of polymers numerically. The initial level quantifies atomic triplets, such as H1–C4–H1, denoting specific atomic configurations. The subsequent level encapsulates predefined chemical building blocks like –C6H4–, –CH2–, and –C(=O)–. The third level encompasses quantitative structure-property relationship (QSPR) descriptors, incorporating molecular features like molecular quantum numbers, molecular connectivity chi indices, non-hydrogen atom count, and molecular weight. These features are then normalized by the number of atoms in the polymer.[4,5]

Molecule fingerprinting follows a similar approach to polymer fingerprinting, involving three hierarchical levels of descriptors. However, unlike polymers, these descriptors are not normalized based on the number of atoms, and certain descriptors like the length of the longest side chain are disregarded. Crucially, ROP specific features, such as the size of the ring being opened and the valence electron differences in the broken bond of the ring, are integrated into the fingerprinting process. These fingerprinting techniques capture the structural information of polymers and molecules, enabling various computational modeling and analysis tasks.[1]

ML Training Space

It is paramount to demonstrate the relationship between the genetic algorithm (GA)-generated polymers against the training space used to train the machine learning (ML) models. To show the training space of the ML model in relation to the GA polymers, we applied Uniform Manifold Approximation and Projection (UMAP)[6] to all data points used for training (shown as orange diamonds) and to the GA data (shown as blue circles). We have divided the UMAP plots based on the different properties being measured: Thermal (decomposition temperature (T_d), glass transition temperature (T_g)), Mechanical (Young's modulus (E),tensile strength at break (σ_b)), Thermodynamic (heat capacity (C_p)) and Enthalpy (ΔH). The latter two properties were separated because different models were used to predict their values. The four green stars in each plot correspond to the four polymers identified in Figure 4d.

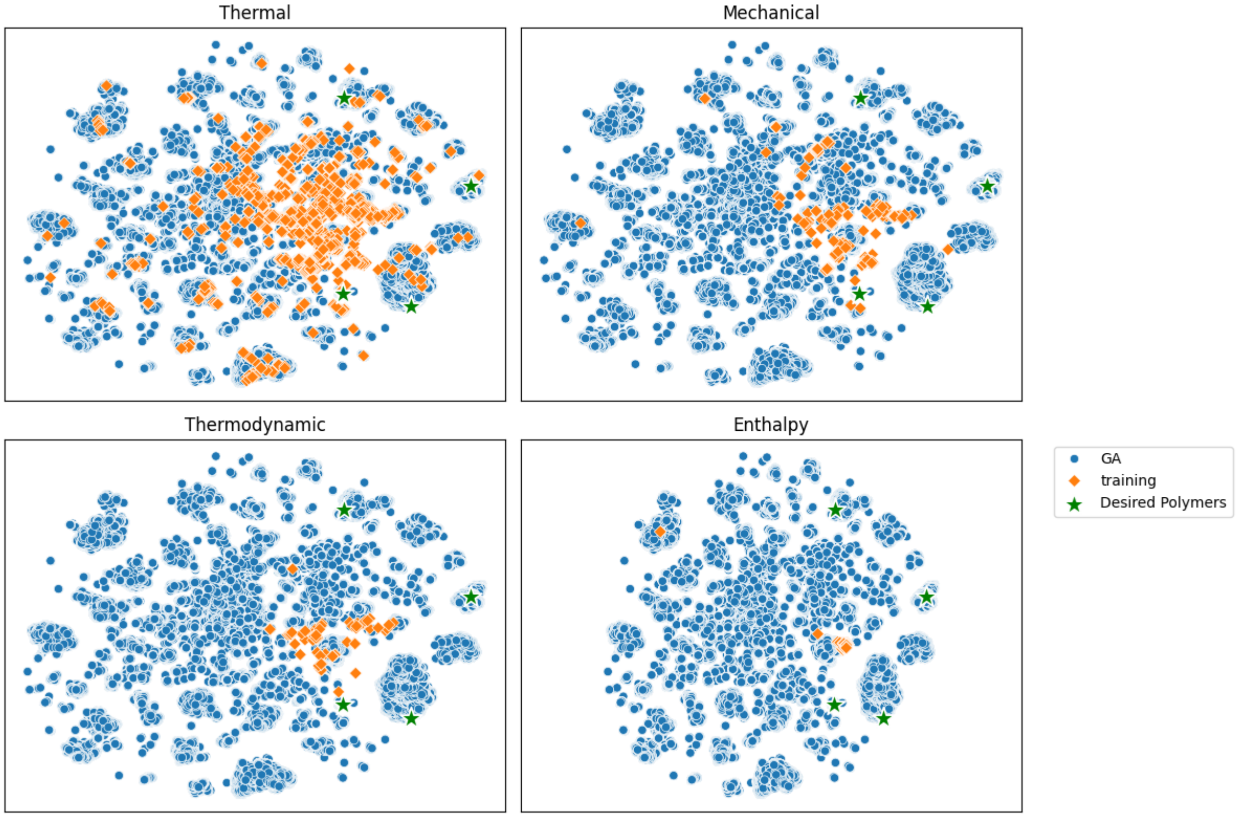

Figure S1: UMAPs demonstrating the training space of the ML model in comparison to the GA polymers. Employing a cosine similarity metric, we configured the UMAP with 200 nearest neighbors and a minimum distance setting of 0.25, optimizing for a balance between local and global manifold structures and ensuring adequate spacing of data points. This is based on the description in previous work on UMAPS on similar polymer space[7]

Our dataset for training thermal property predictions is quite robust, with all target polymers situated close to previously encountered examples. In contrast, the GA ventures into uncharted chemical territory predicting enthalpy, mechanical properties, and thermodynamic properties beyond the scope of the training data. Consequently, model accuracy may be limited for these properties, and significant enhancements can be anticipated as the model training datasets expand to encompass a more extensive chemical space

Scaffold Generation

These scaffolds were meticulously curated through an extensive analysis of a diverse database of known molecules, amalgamated from various reputable sources including ZINC15, ChemBL, compounds extracted from literature, and an eMolecules database snapshot from December 19th, 2020.

To identify suitable scaffolds for each reaction class, we systematically queried the database for molecules exhibiting the substructure shown in the "Reaction" column of Table 3. Following this, we employed RDKit to remove explicit hydrogens and stereochemistry and canonicalize molecules. Unique molecules were then stripped of all side chains, and the result was subsequently organized into a dictionary format where each scaffold's Simplified molecular-input line-entry system (SMILES) notation was mapped to its frequency count.[8]

Our selection process prioritized scaffolds based on their prevalence within the database, operating under the premise that such structures are more representative and feasible to synthesize. For instance, in S2, we showcase the top seven scaffolds for five-member lactone rings as an illustrative example. Subsequently, a subset of these scaffolds was chosen to serve as the foundational framework for esters in our study.

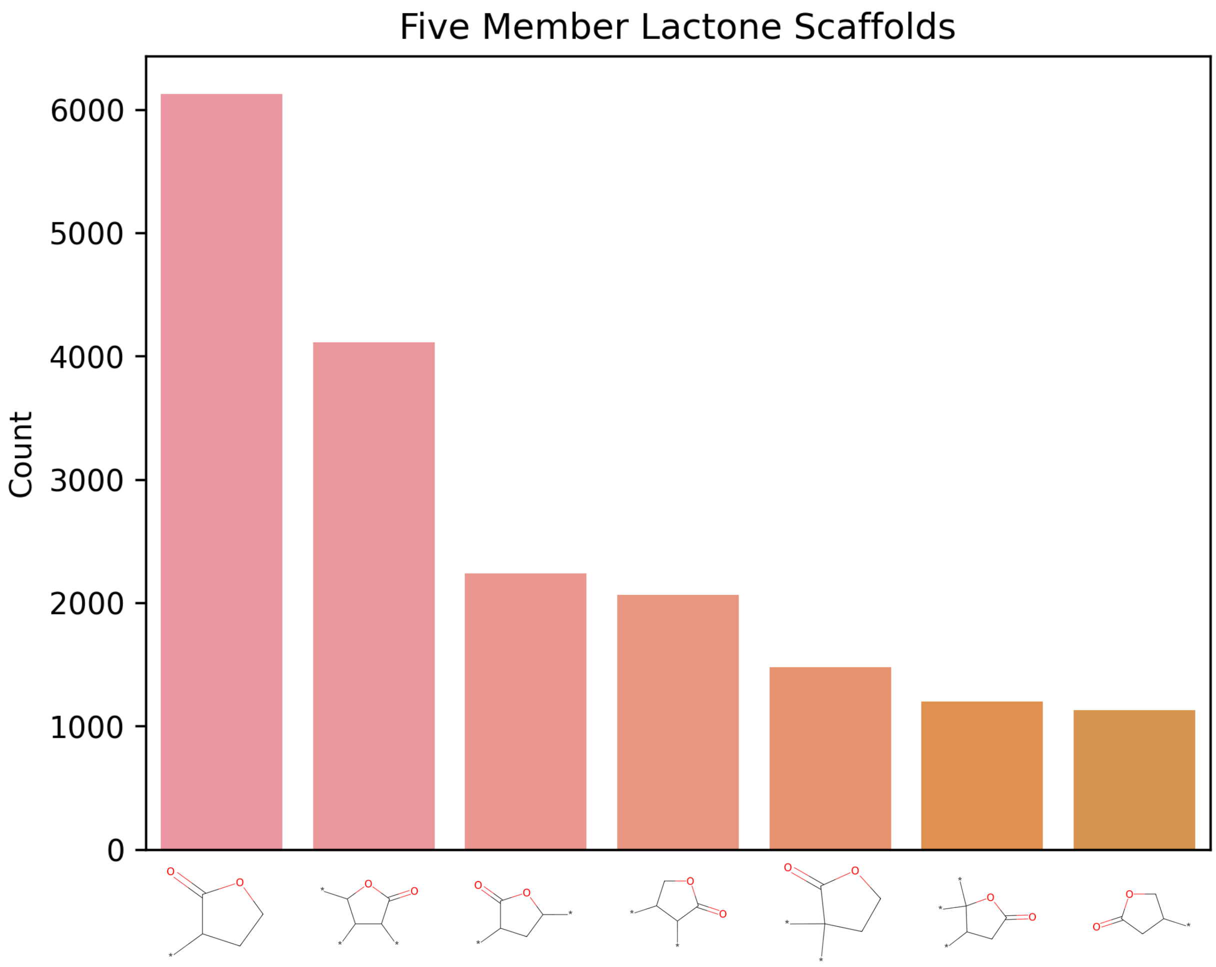

Figure S2: Top seven scaffolds found from a list of 26,883 known five-member lactone ring. Asterisks (*) represent connection points for the R-Groups.

By employing this approach, we identified scaffolds tailored to the reaction classes outlined in Table 3. To reduce the complexity of the resulting molecules, we deliberately selected scaffolds with only one or two available R-group attachment points, displayed in S3

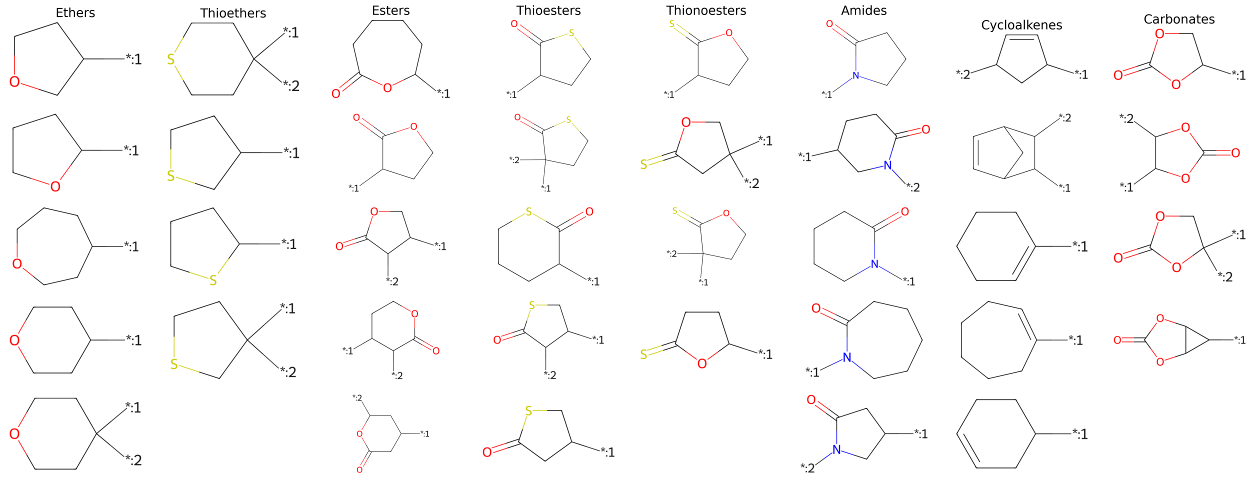

Figure S3: A table of all the classes along with their scaffolds used, chosen according to the methodology described in Section: Scaffold Generation

.

Ethers Case Study

To ensure our GA efficiently identifies promising polymer candidates and is not simply getting lucky, we conducted a case study comparing enumerative results with GA outcomes. We selected scaffolds from the ether column (see Figure S3) and chose 700 R-groups to generate a computationally manageable number of polymers for enumeration (totaling 492,800). To prioritize simpler molecules and those more likely to exhibit all desired properties, we selected 1,400 fragments: 700 with the lowest molecule complexity score (SA) from polymers achieving all properties and 700 from the global R-group list. After removing duplicates, 700 were randomly selected we were left with 297 fragments from the global list and the remaining 403 from top-performing polymers.

Next, we conducted an exhaustive enumeration, systematically generating all possible combinations of R-groups and scaffolds, and subsequently polymerizing and fingerprinting the resulting polymers. We then predicted their properties using our established models. Following this, we executed a single run of the GA as detailed in the main paper's methods section, utilizing the reduced R-group list. The outcomes are visualized in S4 (a) and (b),

which displays histograms of fitness values (calculated as described in the main paper's methods section) for the enumerated polymer space (blue) and the GA run (orange).

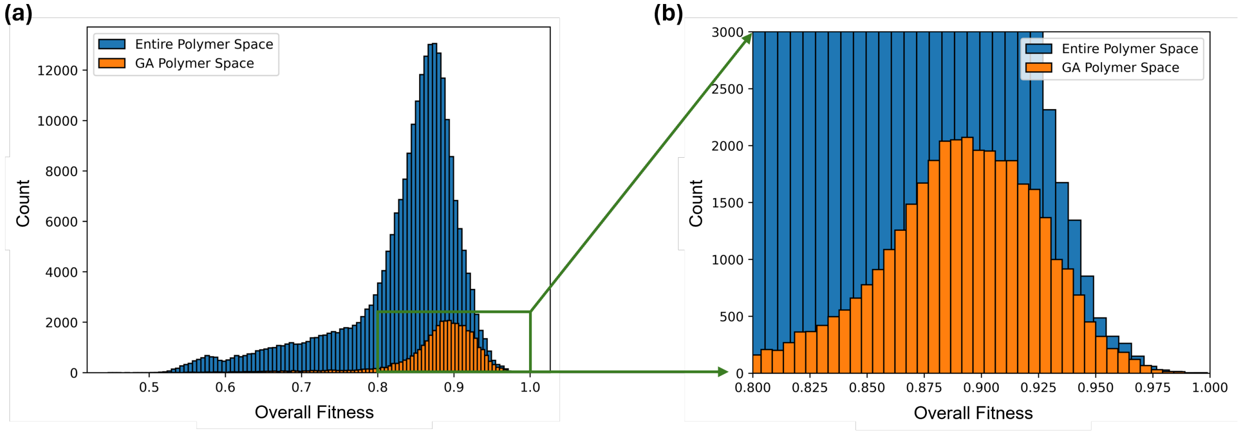

Figure S4: (a) Fitness values for the GA (orange) compared to the fitness values of the enumerated polymer space (blue) for the ether scaffolds with 700 R-groups. (b) Zoomed in view.

The histogram reveals that the GA successfully identifies a significant number of polymers within the desired fitness region, characterized by values close to one. Notably, the GA's distribution is shifted to the right compared to the enumerative distribution, indicating its optimization focus. Moreover, the GA explored a relatively small area of the entire space, covering only approximately 7.8% (38,479 out of 492,800) of the polymers, yet it managed to capture an impressive 79% (975 out of 1241) of the top 0.5% of polymers. This demonstrates that the GA is not merely relying on chance; rather, it effectively targets and identifies the most promising candidates.

However, a common challenge with GAs is their propensity to converge on a narrow molecular space that meets the target properties, potentially limiting their exploratory capacity. To investigate whether these optimized molecules occupy a restricted design space, we conducted principal component analysis (PCA) on the polymer fingerprints and plotted the two most significant components in S5. The results reveal that the molecule space not only covers a substantial portion of the enumerated space but also maintains this diversity even in later generations. This is likely attributed to the enforced mutation scheme, which

introduces variations when the algorithm encounters a polymer more than once, promoting exploration and preventing convergence into a narrow space.

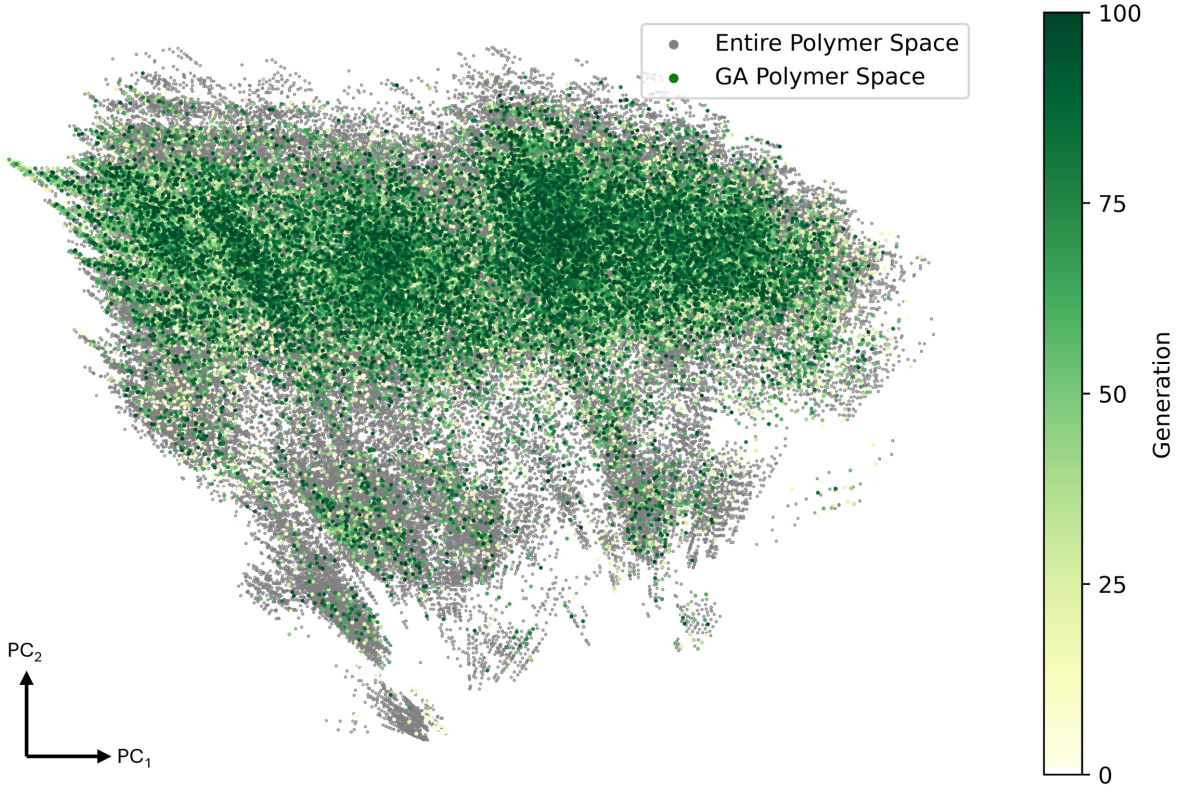

Figure S5: PCA plots of the space The green dots depict polymers explored by the GA. The lightest color denotes the initial generation, while the darkest signifies the 100th and final generation.

Promising Candidates

Figure S6 (a) reveals that scaffolds with two R-group locations dominate the population of polymers achieving all target properties, with only a negligible number of single R-group scaffolds meeting these criteria. In contrast, (b) illustrates the trade-off between molecular complexity and synthesizability, as the distribution of SA scores for monomers with two R-group locations is right-shifted compared to those with one. This comparison highlights that increasing the number of R-groups, while potentially improving property optimization, also increases molecular complexity and reduces the likelihood of successful synthesis. Lastly, (c)

displays the distribution of SA scores for monomers that yield polymers meeting all property criteria, categorized by monomer class. Notably, the synthetic complexity of these molecules, as shown in (c), follows a similar distribution to that in (b), indicating that a significant number of these solutions may be synthetically infeasible, a consequence of the findings in (a).

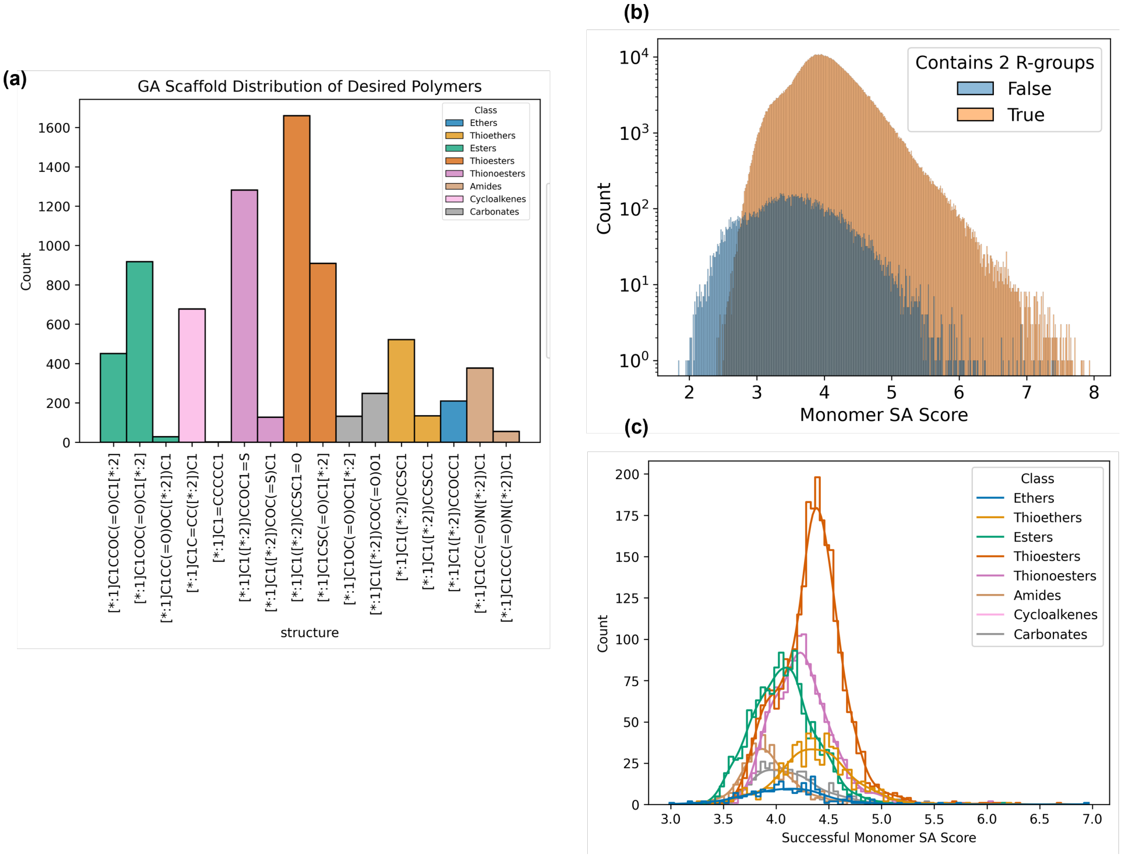

Figure S6: (a) Histogram showing which scaffolds had the most polymers that achieved all properties. (b) Histogram comparing the synthetic accessibility score (SAScore) score of monomers with two R-group locations to those with one. (c) Histogram with kernel density estimate (KDE) overlay displaying the distribution of SAScore scores for monomers resulting in polymers with all target properties.

ROP Class Evolution

This section presents the evolution of all ROP classes for each property tested Figure S7(a). We specifically note that top children of most classes do not attain E and ΔH, potentially

due to the constant mutation of their R-groups. This also helps explain why runs with fewer scaffolds containing 2 R-group sites have lower counts in Figure 3(d) of the main text. This, however, is the average of the 100 top-performing children; the top 10-30 polymers reach the threshold. In figure S7(b), the stagnant nature of the SAScore resembles the difficulty in attaining less complex monomers that could meet our design criteria.

(a)

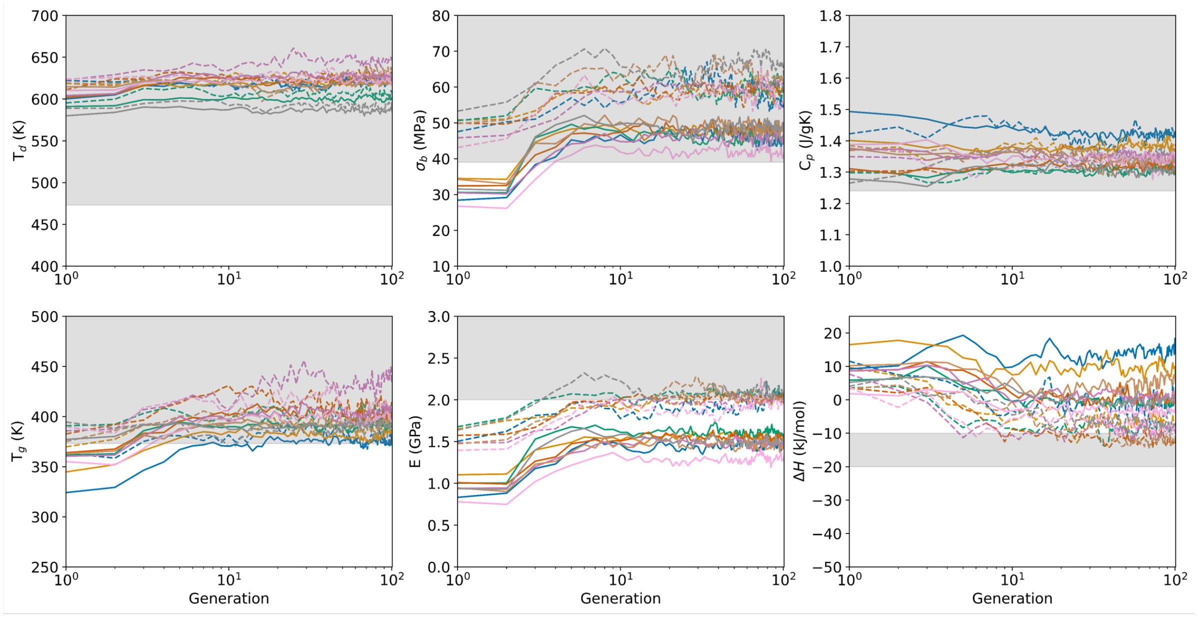

(b)

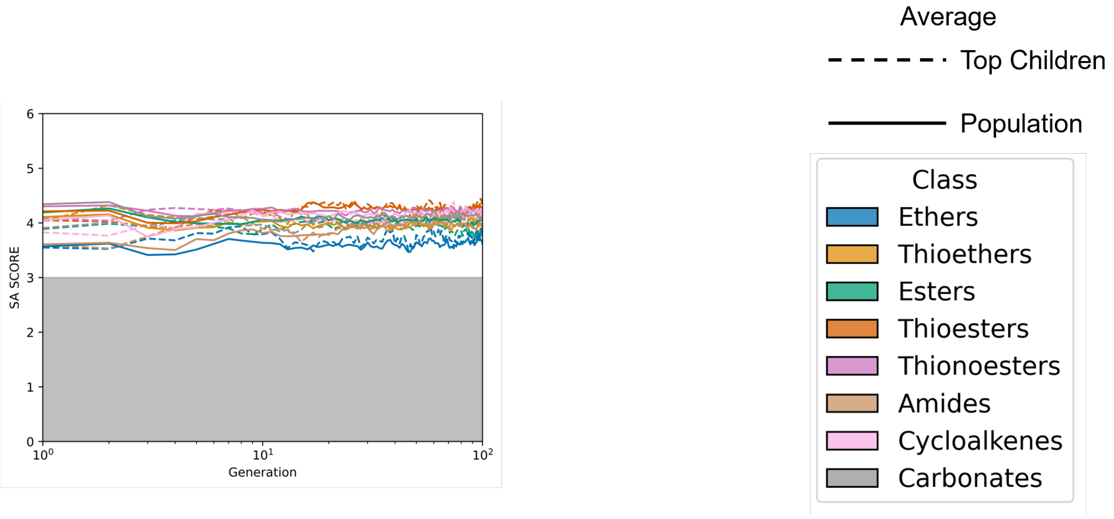

Figure S7: (a) Line plot showing the change in average properties for top children polymers (dashed lines) and the total population (solid lines) over generations for one run of the GA on each class of monomers. The top 100 polymers, selected based on their fitness function values, serve as parents for the next generation. The grey region indicates the target property range. (b) SAScore evolution of top children and overall population for all ROP class monomers.

References

(1) Toland, A.; Tran, H.; Chen, L.; Li, Y.; Zhang, C.; Gutekunst, W.; Ramprasad, R. Accelerated Scheme to Predict Ring-Opening Polymerization Enthalpy: Simulation-Experimental Data Fusion and Multitask Machine Learning. *The Journal of Physical Chemistry A* **2023**, *127*, 10709–10716, Publisher: American Chemical Society.

(2) Strategies to combine ROP with ATRP or RAFT polymerization for the synthesis of biodegradable polymeric nanoparticles for biomedical applications - Polymer Chemistry (RSC Publishing). https://pubs.rsc.org/en/content/articlelanding/2018/py/c8py00649k.

(3) Kuenneth, C.; Ramprasad, R. polyBERT: a chemical language model to enable fully machine-driven ultrafast polymer informatics. *Nature Communications* **2023**, *14*, 4099, Publisher: Nature Publishing Group.

(4) Kim, C.; Chandrasekaran, A.; Huan, T. D.; Das, D.; Ramprasad, R. Polymer Genome: A Data-Powered Polymer Informatics Platform for Property Predictions. *The Journal of Physical Chemistry C* **2018**, *122*, 17575–17585, Publisher: American Chemical Society.

(5) Doan Tran, H.; Kim, C.; Chen, L.; Chandrasekaran, A.; Batra, R.; Venkatram, S.; Kamal, D.; Lightstone, J. P.; Gurnani, R.; Shetty, P.; Ramprasad, M.; Laws, J.; Shelton, M.; Ramprasad, R. Machine-learning predictions of polymer properties with Polymer Genome. *Journal of Applied Physics* **2020**, *128*, 171104.

(6) McInnes, L.; Healy, J.; Saul, N.; Großberger, L. UMAP: Uniform Manifold Approximation and Projection. *Journal of Open Source Software* **2018**, *3*, 861.

(7) Kern, J.; Su, Y.; Gutekunst, W.; Ramprasad, R. An Informatics Framework for the Design of Sustainable, Chemically Recyclable, Synthetically-Accessible and Durable Polymers. 2024; http://arxiv.org/abs/2409.15354, arXiv:2409.15354.

(8) RDKit. https://www.rdkit.org/.
